# *N*-Isopropyl-6-methyl-2-phenyl­quinoline-3-carboxamide

**DOI:** 10.1107/S1600536810031582

**Published:** 2010-08-18

**Authors:** Saida Benzerka, Abdelmalek Bouraiou, Sofiane Bouacida, Thierry Roisnel, Ali Belfaitah

**Affiliations:** a Laboratoire des Produits Naturels d’Origine Végétale et de Synthèse Organique, PHYSYNOR, Université Mentouri-Constantine, 25000 Constantine, Algeria; b Unité de Recherche de Chimie de l’Environnement et Moléculaire Structurale, CHEMS, Université Mentouri-Constantine, 25000 Algeria; c Centre de Difractométrie X, UMR 6226 CNRS Unité Sciences Chimiques de Rennes, Université de Rennes I, 263 Avenue du Général Leclerc, 35042 Rennes, France

## Abstract

In the title compound, C_20_H_20_N_2_O, the dihedral angle between the quinoline ring system and the phenyl ring is 49.40 (5)°. In the crystal structure, zigzag layers of mol­ecules, in which the quinoline units are parallel to the (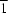
10) plane, are arranged perpendicular to the *b* axis. Inter­molecular N—H⋯O hydrogen bonds connect the mol­ecules into chains along [010], reinforcing the cohesion between the layers of the structure.

## Related literature

For our previous work on the preparation of quinoline deriv­atives, see: Benzerka *et al.* (2008 ▶); Ladraa *et al.* (2009 ▶); Bouraiou *et al.* (2006 ▶, 2008 ▶). For the evaluation of their biological activity, see: Atwell *et al.* (1988 ▶,1989 ▶); Denny *et al.* (1990 ▶); Toshima *et al.* (1999 ▶); Mikata *et al.* (1998 ▶); Henriksen *et al.* (1991 ▶). For the synthetic procedure, see: Saudi *et al.* (2003 ▶).
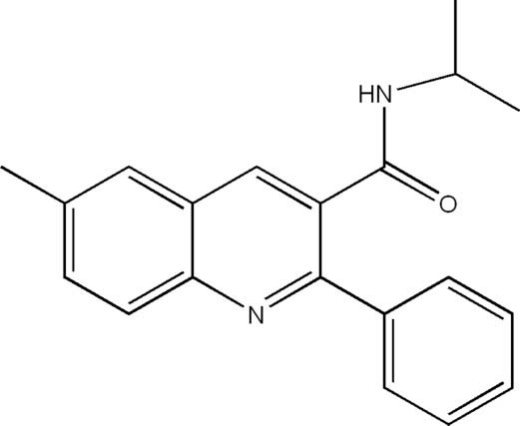


## Experimental

### 

#### Crystal data

C_20_H_20_N_2_O
*M* *_r_* = 304.38
Orthorhombic, 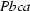

*a* = 12.0007 (3) Å
*b* = 9.6314 (2) Å
*c* = 29.4627 (8) Å
*V* = 3405.40 (14) Å^3^
*Z* = 8
Mo *K*α radiation
μ = 0.07 mm^−1^
*T* = 150 K
0.32 × 0.11 × 0.08 mm

#### Data collection

Bruker APEXII diffractometer
Absorption correction: multi-scan (*SADABS*; Bruker, 2001 ▶) *T*_min_ = 0.747, *T*_max_ = 0.994
15315 measured reflections
3906 independent reflections
2839 reflections with *I* > 2σ(*I*)
*R*_int_ = 0.046

#### Refinement

*R*[*F* ^2^ > 2σ(*F* ^2^)] = 0.050
*wR*(*F* ^2^) = 0.157
*S* = 1.04
3906 reflections
211 parameters
H-atom parameters constrained
Δρ_max_ = 0.23 e Å^−3^
Δρ_min_ = −0.26 e Å^−3^

### 

Data collection: *APEX2* (Bruker, 2001 ▶); cell refinement: *SAINT* (Bruker, 2001 ▶); data reduction: *SAINT*; program(s) used to solve structure: *SIR2002* (Burla *et al.*, 2003 ▶); program(s) used to refine structure: *SHELXL97* (Sheldrick, 2008 ▶); molecular graphics: *ORTEP-3 for Windows* (Farrugia, 1997 ▶) and *DIAMOND* (Brandenburg & Berndt, 2001 ▶); software used to prepare material for publication: *WinGX* publication routines (Farrugia, 1999 ▶).

## Supplementary Material

Crystal structure: contains datablocks I. DOI: 10.1107/S1600536810031582/lh5101sup1.cif

Structure factors: contains datablocks I. DOI: 10.1107/S1600536810031582/lh5101Isup2.hkl

Additional supplementary materials:  crystallographic information; 3D view; checkCIF report

## Figures and Tables

**Table 1:** Hydrogen-bond geometry (Å, °)

*D*—H⋯*A*	*D*—H	H⋯*A*	*D*⋯*A*	*D*—H⋯*A*
N2—H2*N*⋯O1^i^	0.88	1.95	2.804 (3)	164

Symmetry code: (i) 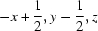.

**Fig. 1.**
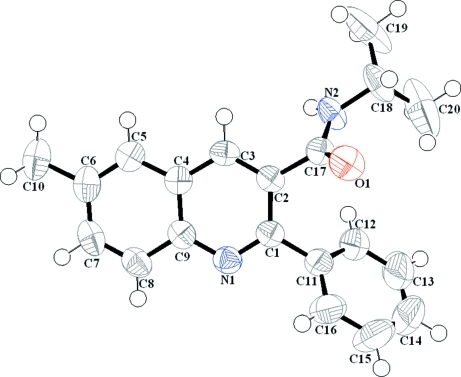
The molecular structure (Farrugia, 1997) of the title compound with the atomic labelling scheme. Displacement are drawn at the 50% probability level.

**Fig. 2.**
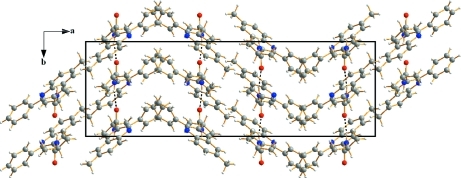
Part of the crystal structure (Brandenburg & Berndt, 2001) showing the layered packing of (I) viewed along the c axis and showing hydrogen bonds [N—H···O] as dashed line along the b axis.

## supplementary crystallographic information

### Comment

Since Atwell *et al.* (1988) and Denny *et al.* (1990) demonstrated the efficacy of 2 + 1 unfused tricyclic aromatic systems such as phenylquinolines as a minimal intercalators, 2-phenylquinoline (Mikata *et al.*, 1998; Henriksen *et al.*, 1991) was selected as the DNA intercalator. The conjugated C=N bond in the 2-phenylquinoline unit was also expected to generate the photoexcited ^3^(n→π*) state upon photoirradiation, which may have a radical character and could be capable of cleaving DNA (Toshima *et al.*, 1999). On the other hand, certain 2-phenylquinoline carboxamide derivatives have been shown to possess DNA binding capability and a broad-spectrum activity in both leukemia and solid-tumor assays (Atwell *et al.*, 1989). As part of our program related to the synthesis of some new heterocyclic compounds with medicinal potential (Bouraiou *et al.*,2006, 2008; Benzerka *et al.*, 2008; Ladraa *et al.*, 2009), we report here the synthesis and crystal structure of the title compound (I). The molecular geometry and the atom-numbering scheme of (I) are shown in Fig. 1. The asymmetric unit of title compound contains a quinolyl unit bearing a phenyl ring at position C-2, amide group at C-3 and methyl at C-6. The two rings of the quinolyl moiety are fused in an axial fashion and form a dihedral angle of 3.13 (4)°. The dihedral angle between the phenyl ring quinoline ring system is 49.40 (5)°. The amide group is essentially planar. The r.m.s deviation for atoms C2/C17/O1/N2/C18 is 0.007Å and the maximum deviation is -0.0131 (15)Å for C17. The C—N [1.3260 (17) Å] bond length to the carbonyl group is closer to that of a standard C═N double bond (1.27 Å) than to that of a single bond (1.49 Å). This is because the lone pair electrons on nitrogen of the amide are delocalized into the carbonyl group. The crystal packing can be described as layers in zig zag perpendicular to *b* axis which quinoline rings are parallel to the (-110) plane (Fig. 2). The crystal packing is stabilized by intermolecular hydrogen bond (N—H···O), resulting in the formation of infinite one-dimensional chain along the *b* axis linked these layers reinforce the cohesion of the structure (Fig. 2).

### Experimental

Compound (I) was obtained from 6-methyl-2-phenylquinoline-3-carboxylic acid and ethyl chloroformate in presence triethylamine in chloroform (Saudi *et al.*, 2003). Suitable crystals for X-ray diffraction were obtained by slow evaporation of a solution of (I) in diisopropylether at room temperature.

### Refinement

All H atoms were located from Fourier maps but introduced in calculated positions and treated as riding on their parent C atom with C-H = 0.93-0.98Å and U_iso_(H) = 1.2U_eq_(C) or 1.5U_eq_(C_methyl_).

### Crystal data

**Table:**

C_20_H_20_N_2_O	*F*(000) = 1296
*M**_r_* = 304.38	*D*_x_ = 1.187 Mg m^−^^3^
Orthorhombic, *P**b**c**a*	Mo *K*α radiation, λ = 0.71073 Å
Hall symbol: -P 2ac 2ab	Cell parameters from 4726 reflections
*a* = 12.0007 (3) Å	θ = 3.0–27.3°
*b* = 9.6314 (2) Å	µ = 0.07 mm^−^^1^
*c* = 29.4627 (8) Å	*T* = 150 K
*V* = 3405.40 (14) Å^3^	Stick, colourless
*Z* = 8	0.32 × 0.11 × 0.08 mm

### Data collection

**Table:**

Bruker APEXII diffractometer	2839 reflections with *I* > 2σ(*I*)
graphite	*R*_int_ = 0.046
CCD rotation images, thin slices scans	θ_max_ = 27.5°, θ_min_ = 2.8°
Absorption correction: multi-scan (*SADABS*; Bruker, 2001)	*h* = −10→15
*T*_min_ = 0.747, *T*_max_ = 0.994	*k* = −7→12
15315 measured reflections	*l* = −24→38
3906 independent reflections	

### Refinement

**Table:**

Refinement on *F*^2^	Primary atom site location: structure-invariant direct methods
Least-squares matrix: full	Secondary atom site location: difference Fourier map
*R*[*F*^2^ > 2σ(*F*^2^)] = 0.050	Hydrogen site location: inferred from neighbouring sites
*wR*(*F*^2^) = 0.157	H-atom parameters constrained
*S* = 1.04	*w* = 1/[σ^2^(*F*_o_^2^) + (0.0914*P*)^2^ + 0.3352*P*] where *P* = (*F*_o_^2^ + 2*F*_c_^2^)/3
3906 reflections	(Δ/σ)_max_ = 0.001
211 parameters	Δρ_max_ = 0.23 e Å^−^^3^
0 restraints	Δρ_min_ = −0.26 e Å^−^^3^

### Special details

**Table:**

Geometry. All e.s.d.'s (except the e.s.d. in the dihedral angle between two l.s. planes) are estimated using the full covariance matrix. The cell e.s.d.'s are taken into account individually in the estimation of e.s.d.'s in distances, angles and torsion angles; correlations between e.s.d.'s in cell parameters are only used when they are defined by crystal symmetry. An approximate (isotropic) treatment of cell e.s.d.'s is used for estimating e.s.d.'s involving l.s. planes.
Refinement. Refinement of *F*^2^ against ALL reflections. The weighted *R*-factor *wR* and goodness of fit *S* are based on *F*^2^, conventional *R*-factors *R* are based on *F*, with *F* set to zero for negative *F*^2^. The threshold expression of *F*^2^ > σ(*F*^2^) is used only for calculating *R*-factors(gt) *etc*. and is not relevant to the choice of reflections for refinement. *R*-factors based on *F*^2^ are statistically about twice as large as those based on *F*, and *R*- factors based on ALL data will be even larger.

### Fractional atomic coordinates and isotropic or equivalent isotropic displacement parameters (Å^2^)

**Table:**

	*x*	*y*	*z*	*U*_iso_*/*U*_eq_	
O1	0.29293 (9)	−0.22115 (10)	0.10660 (4)	0.0378 (3)	
N1	0.58409 (10)	−0.43666 (12)	0.14988 (4)	0.0288 (3)	
N2	0.21063 (10)	−0.43091 (12)	0.09977 (4)	0.0309 (3)	
H2N	0.2211	−0.5213	0.0984	0.037*	
C1	0.47981 (11)	−0.39345 (14)	0.14808 (5)	0.0258 (3)	
C2	0.40933 (11)	−0.41975 (13)	0.10973 (5)	0.0240 (3)	
C3	0.44903 (12)	−0.50023 (13)	0.07517 (5)	0.0249 (3)	
H3	0.4038	−0.5201	0.0504	0.030*	
C4	0.55847 (12)	−0.55334 (13)	0.07693 (4)	0.0251 (3)	
C5	0.60463 (12)	−0.64177 (14)	0.04333 (5)	0.0289 (3)	
H5	0.5619	−0.6654	0.0182	0.035*	
C6	0.71088 (13)	−0.69338 (15)	0.04705 (5)	0.0335 (4)	
C7	0.77548 (13)	−0.65292 (17)	0.08495 (6)	0.0352 (4)	
H7	0.8477	−0.6870	0.0878	0.042*	
C8	0.73505 (13)	−0.56529 (16)	0.11750 (5)	0.0332 (4)	
H8	0.7805	−0.5384	0.1415	0.040*	
C9	0.62453 (11)	−0.51521 (14)	0.11490 (5)	0.0266 (3)	
C10	0.75887 (16)	−0.79114 (18)	0.01255 (6)	0.0448 (4)	
H10A	0.7119	−0.7928	−0.0138	0.067*	
H10B	0.7633	−0.8827	0.0253	0.067*	
H10C	0.8321	−0.7604	0.0041	0.067*	
C11	0.43566 (13)	−0.32106 (16)	0.18883 (5)	0.0328 (4)	
C12	0.33582 (14)	−0.3632 (2)	0.20819 (6)	0.0464 (5)	
H12	0.2956	−0.4352	0.1950	0.056*	
C13	0.29535 (17)	−0.2991 (3)	0.24697 (7)	0.0681 (7)	
H13	0.2292	−0.3291	0.2602	0.082*	
C14	0.3541 (2)	−0.1903 (3)	0.26577 (8)	0.0792 (8)	
H14	0.3264	−0.1452	0.2913	0.095*	
C15	0.4537 (2)	−0.1480 (2)	0.24698 (7)	0.0690 (7)	
H15	0.4928	−0.0746	0.2599	0.083*	
C16	0.49557 (16)	−0.21433 (18)	0.20900 (6)	0.0454 (4)	
H16	0.5639	−0.1874	0.1970	0.055*	
C17	0.29824 (12)	−0.34913 (13)	0.10575 (5)	0.0258 (3)	
C18	0.09700 (12)	−0.37692 (16)	0.09527 (6)	0.0395 (4)	
H18	0.1017	−0.2852	0.0810	0.047*	
C19	0.03298 (16)	−0.4717 (2)	0.06337 (11)	0.0859 (9)	
H19A	0.0293	−0.5633	0.0762	0.129*	
H19B	0.0703	−0.4755	0.0346	0.129*	
H19C	−0.0411	−0.4363	0.0592	0.129*	
C20	0.04341 (19)	−0.3592 (3)	0.14068 (8)	0.0850 (9)	
H20A	0.0880	−0.2983	0.1590	0.128*	
H20B	0.0374	−0.4479	0.1553	0.128*	
H20C	−0.0296	−0.3201	0.1369	0.128*	

### Atomic displacement parameters (Å^2^)

**Table:**

	*U*^11^	*U*^22^	*U*^33^	*U*^12^	*U*^13^	*U*^23^
O1	0.0403 (7)	0.0158 (5)	0.0574 (7)	−0.0006 (4)	−0.0080 (5)	0.0015 (4)
N1	0.0269 (6)	0.0314 (6)	0.0281 (6)	−0.0029 (5)	0.0000 (5)	0.0012 (5)
N2	0.0234 (6)	0.0155 (5)	0.0539 (8)	0.0019 (5)	−0.0045 (6)	0.0033 (5)
C1	0.0264 (7)	0.0220 (6)	0.0290 (7)	−0.0029 (5)	−0.0008 (6)	0.0020 (5)
C2	0.0241 (7)	0.0167 (6)	0.0312 (7)	−0.0031 (5)	−0.0019 (6)	0.0030 (5)
C3	0.0287 (7)	0.0185 (6)	0.0276 (7)	−0.0038 (5)	−0.0045 (6)	0.0018 (5)
C4	0.0285 (7)	0.0194 (6)	0.0276 (7)	−0.0035 (6)	0.0024 (6)	0.0046 (5)
C5	0.0338 (8)	0.0236 (7)	0.0293 (7)	−0.0017 (6)	0.0025 (6)	0.0039 (6)
C6	0.0369 (9)	0.0269 (7)	0.0367 (8)	0.0015 (6)	0.0116 (7)	0.0071 (6)
C7	0.0260 (8)	0.0376 (8)	0.0421 (9)	0.0049 (6)	0.0069 (7)	0.0106 (7)
C8	0.0260 (8)	0.0407 (9)	0.0328 (8)	−0.0018 (6)	−0.0001 (6)	0.0064 (6)
C9	0.0260 (7)	0.0254 (7)	0.0283 (7)	−0.0030 (6)	0.0024 (6)	0.0066 (6)
C10	0.0501 (10)	0.0400 (9)	0.0444 (10)	0.0111 (8)	0.0159 (8)	0.0012 (8)
C11	0.0324 (8)	0.0356 (8)	0.0304 (8)	0.0071 (7)	−0.0068 (6)	−0.0031 (6)
C12	0.0364 (10)	0.0642 (12)	0.0387 (10)	0.0050 (8)	0.0019 (7)	−0.0107 (8)
C13	0.0471 (12)	0.112 (2)	0.0449 (11)	0.0199 (12)	0.0061 (9)	−0.0181 (12)
C14	0.0741 (16)	0.115 (2)	0.0487 (13)	0.0337 (15)	−0.0048 (12)	−0.0389 (13)
C15	0.0828 (17)	0.0685 (14)	0.0557 (13)	0.0135 (12)	−0.0237 (12)	−0.0327 (11)
C16	0.0486 (10)	0.0439 (10)	0.0437 (10)	0.0031 (8)	−0.0122 (8)	−0.0103 (8)
C17	0.0289 (8)	0.0179 (6)	0.0307 (7)	0.0003 (5)	−0.0024 (6)	0.0015 (5)
C18	0.0243 (8)	0.0248 (7)	0.0695 (11)	0.0047 (6)	−0.0029 (8)	0.0120 (7)
C19	0.0369 (11)	0.0361 (10)	0.185 (3)	0.0037 (8)	−0.0494 (14)	−0.0047 (14)
C20	0.0510 (13)	0.112 (2)	0.0926 (18)	0.0396 (13)	0.0298 (12)	0.0511 (16)

### Geometric parameters (Å, °)

**Table:**

O1—C17	1.2346 (16)	C10—H10B	0.9600
N1—C1	1.3198 (18)	C10—H10C	0.9600
N1—C9	1.3676 (18)	C11—C12	1.388 (2)
N2—C17	1.3255 (18)	C11—C16	1.388 (2)
N2—C18	1.4654 (18)	C12—C13	1.387 (3)
N2—H2N	0.8800	C12—H12	0.9300
C1—C2	1.4339 (19)	C13—C14	1.379 (4)
C1—C11	1.486 (2)	C13—H13	0.9300
C2—C3	1.3656 (19)	C14—C15	1.378 (4)
C2—C17	1.5012 (19)	C14—H14	0.9300
C3—C4	1.4104 (19)	C15—C16	1.383 (3)
C3—H3	0.9300	C15—H15	0.9300
C4—C5	1.4187 (19)	C16—H16	0.9300
C4—C9	1.419 (2)	C18—C20	1.494 (3)
C5—C6	1.373 (2)	C18—C19	1.519 (3)
C5—H5	0.9300	C18—H18	0.9800
C6—C7	1.414 (2)	C19—H19A	0.9600
C6—C10	1.501 (2)	C19—H19B	0.9600
C7—C8	1.366 (2)	C19—H19C	0.9600
C7—H7	0.9300	C20—H20A	0.9600
C8—C9	1.413 (2)	C20—H20B	0.9600
C8—H8	0.9300	C20—H20C	0.9600
C10—H10A	0.9600		
			
C1—N1—C9	118.72 (12)	C12—C11—C1	120.19 (14)
C17—N2—C18	122.63 (11)	C16—C11—C1	120.57 (15)
C17—N2—H2N	118.7	C13—C12—C11	120.73 (19)
C18—N2—H2N	118.7	C13—C12—H12	119.6
N1—C1—C2	122.37 (13)	C11—C12—H12	119.6
N1—C1—C11	116.96 (12)	C14—C13—C12	119.3 (2)
C2—C1—C11	120.61 (12)	C14—C13—H13	120.3
C3—C2—C1	118.81 (13)	C12—C13—H13	120.3
C3—C2—C17	120.58 (12)	C13—C14—C15	120.4 (2)
C1—C2—C17	120.36 (12)	C13—C14—H14	119.8
C2—C3—C4	120.22 (13)	C15—C14—H14	119.8
C2—C3—H3	119.9	C14—C15—C16	120.2 (2)
C4—C3—H3	119.9	C14—C15—H15	119.9
C3—C4—C5	123.75 (13)	C16—C15—H15	119.9
C3—C4—C9	117.09 (12)	C15—C16—C11	120.01 (19)
C5—C4—C9	119.16 (13)	C15—C16—H16	120.0
C6—C5—C4	121.61 (13)	C11—C16—H16	120.0
C6—C5—H5	119.2	O1—C17—N2	123.72 (13)
C4—C5—H5	119.2	O1—C17—C2	119.78 (12)
C5—C6—C7	118.20 (14)	N2—C17—C2	116.46 (11)
C5—C6—C10	121.96 (15)	N2—C18—C20	111.09 (14)
C7—C6—C10	119.83 (15)	N2—C18—C19	108.26 (13)
C8—C7—C6	121.98 (14)	C20—C18—C19	113.9 (2)
C8—C7—H7	119.0	N2—C18—H18	107.8
C6—C7—H7	119.0	C20—C18—H18	107.8
C7—C8—C9	120.40 (14)	C19—C18—H18	107.8
C7—C8—H8	119.8	C18—C19—H19A	109.5
C9—C8—H8	119.8	C18—C19—H19B	109.5
N1—C9—C8	118.75 (13)	H19A—C19—H19B	109.5
N1—C9—C4	122.61 (13)	C18—C19—H19C	109.5
C8—C9—C4	118.59 (13)	H19A—C19—H19C	109.5
C6—C10—H10A	109.5	H19B—C19—H19C	109.5
C6—C10—H10B	109.5	C18—C20—H20A	109.5
H10A—C10—H10B	109.5	C18—C20—H20B	109.5
C6—C10—H10C	109.5	H20A—C20—H20B	109.5
H10A—C10—H10C	109.5	C18—C20—H20C	109.5
H10B—C10—H10C	109.5	H20A—C20—H20C	109.5
C12—C11—C16	119.20 (16)	H20B—C20—H20C	109.5

### Hydrogen-bond geometry (Å, °)

**Table:**

*D*—H···*A*	*D*—H	H···*A*	*D*···*A*	*D*—H···*A*
N2—H2N···O1^i^	0.88	1.95	2.804 (3)	164.

Symmetry codes: (i) −*x*+1/2, *y*−1/2, *z*.
